# Damage Behaviour of Shot-Peened 7075 Aluminium Alloy Based on Temperature Evolution and Digital Image Correlation Methods

**DOI:** 10.3390/ma18143228

**Published:** 2025-07-08

**Authors:** Yutong Tang, Aifeng Jiang, Lei Li, Yanliang Dong, Le Chang

**Affiliations:** School of Science, Inner Mongolia University of Technology, Aimin Sub-District, Xincheng District, Hohhot 010051, China; tyt1527497294@163.com (Y.T.); leillt@163.com (L.L.); 13012775679@163.com (Y.D.); 13270300700@163.com (L.C.)

**Keywords:** 7075 aluminium alloy, shot peening, damage evolution, digital image correlation method, temperature evolution

## Abstract

The peening process plays a pivotal role in enhancing the properties of aluminium alloys across various industries, including aerospace, automotive, and construction. Among the critical factors influencing this process, the shot peening time is of paramount importance for studying material characteristics. In the present study, we undertook a comprehensive investigation into the mechanical properties, surface roughness, and damage evolution behaviour of 7075 aluminium alloy subjected to different shot peening durations. This investigation was conducted using a microhardness tester, laser confocal microscope, scanning electron microscope, and other advanced equipment, in conjunction with digital image correlation methods and temperature evolution analysis. Our findings demonstrate that the shot peening time has a profound impact on the mechanical properties of the 7075 alloy. Specifically, the microhardness, tensile strength, and surface roughness of the alloy increased with increasing shot peening time, whereas the elongation rate exhibited a non-monotonic trend, initially decreasing and then increasing. Utilising DIC and temperature evolution analysis, we analysed the influence of shot peening time on the damage evolution behaviour of the alloy and developed tensile damage evolution equations tailored to different shot peening durations. The damage evolution of the 7075 alloy under various shot peening times was observed to proceed through two distinct stages: smooth development and rapid damage. Notably, the damage evolution laws derived from both techniques exhibited good consistency and agreement. The present study serves as a theoretical foundation for exploring the surface peening and damage evolution of 7075 aluminium alloy, which holds significant implications for optimising peening parameters and predicting material life in engineering applications.

## 1. Introduction

Owing to its superior specific strength, low density, exceptional corrosion resistance, and favorable fabrication characteristics, 7075 aluminium alloy (Al-Cu-Mg-Zn system alloy) finds extensive employment in aerospace frameworks, automotive structural components, and civil engineering applications [1,2,3]. Surface peening processes are often used in engineering to improve the properties, service performance, and service life of aluminium alloys. Among the many surface peening processes, shot peening technology has become a widely used surface treatment method for metallic materials owing to its unique advantages. It can refine the microstructure without changing the chemical composition of the material while simultaneously achieving a strengthening effect [4,5,6]. During the shot peening process, a series of changes occur in the internal organisation and structure of the material. These changes are the basis of the subsequent damage evolution, which is directly related to the stability of the material’s performance and life in actual use. Therefore, the study of the damage evolution of metal materials has important theoretical and practical significance, not only for enhancing the reliability and performance of metal materials but also for the positive impact on engineering design, accident prevention, economic efficiency, sustainable development, and other aspects [7].

Contemporary investigations into aluminium alloy surface enhancement have prioritised understanding process parameter effects on mechanical characteristics. Consequently, comprehensive examinations have methodically scrutinised critical variables governing media impact treatment outcomes (e.g., projectile size, impact velocity, spray angle, shot peening time, and coverage) on the properties of the material surface, such as hardness, roughness, and residual stresses [8,9,10]. L Trsko [11] and others investigated the effects of different shot peening strengths on the fatigue life and number of cracks in 7075 aluminium alloy and found that shot peening can improve the fatigue life and number of cracks in the material, but when the shot-peening strength is too high, it will significantly reduce the fatigue life of the aluminium alloy. Wang Mai et al. [12] analysed the effect of shot peening on the corrosion resistance of 7075 aluminium alloy using ultrasonic shot peening technology, and the results showed that ultrasonic shot peening treatment improved the corrosion resistance of the aluminium alloy under the same salt spray corrosive environment, and increasing the power of ultrasonic shot peening within a certain range could further improve its corrosion resistance. Jagannati et al. [13] investigated the effects of different shot peening times on the mechanical properties of aluminium alloys, and the experimental results showed that the shot-peened aluminium alloys had lower elongation and higher surface roughness. Kai Liao et al. [14] investigated the effects of different shot peening coverages on the surface integrity of 7075 aluminium alloy by means of stress tests, hardness tests, scanning electron microscope observations, and surface roughness measurements. The results showed that the surface integrity of the material was improved with the increase in the coverage rate, which helped to maintain the shape stability of the alloy components. Abeens et al. [15] compared and analysed the frictional wear behaviour before and after shot peening, and the results showed that the hardness and roughness of the alloys were significantly improved after shot peening, and the adhesive wear was significantly reduced. Vázquez et al. [16] studied the effects of shot peening and laser peening on the fatigue life of micromanipulators, and the results showed that both treatments were effective, but shot peening was more effective.

In practical engineering applications, components or structures composed of 7075 aluminium alloy are often subjected to a variety of complex loads and environmental factors, so real-time and accurate full-field monitoring of the damage evolution process is crucial. Marcos et al. [17] analysed the microstructure of the material through intermittent monotonic tensile loading tests combined with acoustic emission techniques and established a damage evolution model. The results showed that the acoustic emission technique is important in quantifying the damage development of 7075 aluminium alloy. Renuka et al. [18] evaluated the microstructure of corroded 7075 aluminium alloy using X-ray tomography and tensile testing and analysed the corrosion damage areas in conjunction with nanoindentation and EDS techniques, which in turn were modelled to better understand the corrosion process of the 7075 alloy. Xiang et al. [19] proposed a simplified probabilistic damage tolerance analysis model, and the results showed good agreement between model predictions and experimental data under different shot peening treatment conditions. Guo Yupei et al. [20] used repeated impact experiments to establish the damage evolution equation of 7075-T7351 aluminium alloy, studied the damage evolution law, and analysed and compared the damage equation. Precipitation-hardening aluminium alloys are widely used in the aerospace industry, which has led to higher demands for characterising their damage evolution mechanisms under complex loads. Traditional techniques such as fluidised bed deposition can achieve surface nanocrystalline grain refinement through low-speed deposition [21].

To capture the details of deformation during damage accurately, digital image correlation (DIC) technology is used to measure the displacement and strain fields on the surface of the deformed specimens [22]. Howook et al. [23] used the DIC technique to study the evolution of the tensile strain distribution of 7075 alloy in different states, and the results showed that the work-hardening rate of 7075 alloy treated by aging for 20 min was higher than that of 7075 alloy treated by aging for 240 min. Florando et al. [24] utilised three-dimensional image correlation and a series of thermocouples to measure the full-field strain and temperature, respectively. Yang Hongfan et al. [25] successfully predicted the dynamic stresses in the adhesive layer of metal–plastic and the linear displacement of adhesive bonded structures during curing and thermal warping by means of sequential surface deformation images obtained using a customised DIC system. However, the DIC technique focuses mainly on mechanical deformation measurements and is deficient in reflecting the energy changes within the material due to damage. Infrared thermal imaging technology (IRT) is a powerful non-destructive evaluation tool that can be effectively used for defect detection in materials such as aluminium alloys and metal matrix composites, and it can be used to study energy dissipation with the help of infrared measurement equipment [26,27]. Kumar et al. [28] used a finite element simulation to predict the temperature distribution of friction stir welded AA7075 workpieces, and the results were in good agreement with the temperature distribution captured by the infrared imager. Hu Yuting [29] comparatively analysed the temperature change characteristics of TC4 titanium alloy specimens under the same tensile and compressive loading rates through uniaxial tensile and compression experiments combined with infrared thermal imaging. In summary, the existing damage assessment methods often rely on a single technology, making it difficult to comprehensively capture the damage information of materials under complex working conditions. Although acoustic emission technology and X-ray tomography can quantify damage development, they cannot visualise material surface deformation and instead focus on microstructure assessment; however, it is difficult to track the dynamic change process of damage in real time. Their static process characteristics impose limitations: they cannot capture the thermo-mechanical coupling response under dynamic loads in situ; the correlation between modification effects and service performance relies on destructive testing. Therefore, this study combines digital image correlation (DIC) and infrared thermal imaging (IRT) technologies. The combination of these two technologies can provide rich information based on complete kinematic and thermal characterisation, furthering our understanding of material behaviour [15,30].

This study combines digital image correlation (DIC) technology and infrared thermal imaging technology (IRT) to achieve high-precision measurement of surface displacement and strain fields on material surfaces while capturing deformation details in real time during the damage process. DIC technology focuses on mechanical deformation measurement, while IRT technology enables non-contact real-time monitoring of changes in material dissipation temperature, providing a powerful tool for comprehensive and in-depth research into material damage evolution processes. This multi-technology integration approach not only enhances the accuracy and comprehensiveness of damage assessment but also provides a theoretical foundation for optimising shot peening parameters and predicting the service life of 7075 aluminium alloy in engineering applications.

This study adopted mechanical shot peening for the surface treatment of 7075 aluminium alloy and comparatively analysed the effects of different shot peening times on the microhardness and surface roughness of the specimens. Based on DIC and IRT, uniaxial tensile experiments were conducted on 7075 aluminium alloy specimens treated with different shot peening times to analyse their mechanical properties, explore the influence of the shot peening time on the damage evolution law of the material, and establish a damage evolution equation.

## 2. Materials and Methods

7075-O aluminium alloy was selected for the experiment, and its chemical composition is presented in Table 1.

**Figure 1:**
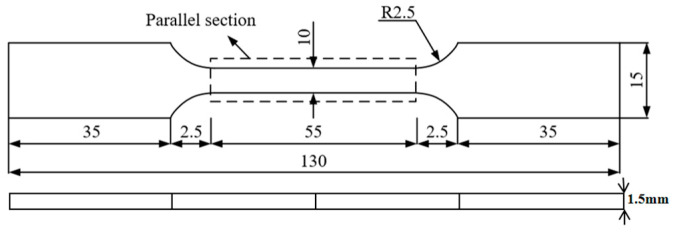
Tensile specimen.

Tensile specimens were prepared from 7075 aluminium alloy sheets of 300 mm × 200 mm using a wire cutting machine, and the specific dimensions of the specimens are shown in Figure 1.

To ensure the surface finish and flatness of the specimens, 400#, 600#, 1000#, and 1500# sandpapers were used in turn for sanding, and the specimens were cleaned utilising an ultrasonic cleaning treatment to make the surface flat and smooth. To investigate the effects of different shot peening times on the mechanical properties and microstructure of the 7075 aluminium alloy, the specimens were shot-peened at room temperature using a Surface Nanochemical Testing Machine for Metallic Materials (SNC-1). SNC-1 uses 306 L stainless steel with a grain size of 3 mm and a frequency of 55 Hz. The shot peening times were 0, 5, 10, and 15 min with 100% coverage and an impingement angle of 90 degrees.

To ensure data reliability, each shot peening condition (0 min, 5 min, 10 min, and 15 min) was tested in triplicate (n = 3). Stress–strain curves were recorded, and statistical analysis was performed to calculate mean values and 95% confidence intervals at key strain points.

The tensile specimens prepared by wire cutting exhibited inherent dimensional variations, as shown in Table 2: the initial thickness of the four groups of specimens, which were not shot-peened (0 min), was distributed within the range of 1.50–1.52 mm (with a measurement deviation of ±0.02 mm). This thickness variation of ±0.65% originated from the systematic errors in the cutting process and was unrelated to subsequent shot peening experiments.

Microhardness tests were performed on the specimens using a semiautomatic digital microhardness tester (AHVD-1000XY, Shanghai Jvjing Precision Instrument Manufacturing Co., Ltd., Shanghai, China). For each specimen, ten hardness test points were selected at random, and the resulting data were statistically analysed to compute the average hardness values. In addition, a laser confocal microscope (LSCM-OLS4100, Olympus, Tokyo, Japan) was used to examine the three-dimensional morphology of the shot-peened surfaces, facilitating the assessment of alterations in surface roughness.

Using the MTS-Landmark 100 kN universal testing machine, strictly following the ISO 6892-1:2019 standard [31], we conducted an axial tensile test with strain rate control (Method A) using a displacement control rate of 3 mm/min. A combination of DIC and IRT was employed to capture the real-time strain and temperature fields on the surface of the specimen during tensile testing. This approach allowed for a comprehensive analysis of the damage evolution of the 7075 aluminium alloy.

Tensile fractures were captured using a field-emission scanning electron microscope (FEI QUANTA 650, FEI, Hillsboro, OR, USA) and subsequently analysed for fracture morphology and characterisation.

## 3. Results

### 3.1. Mechanical Properties

**Figure 2:**
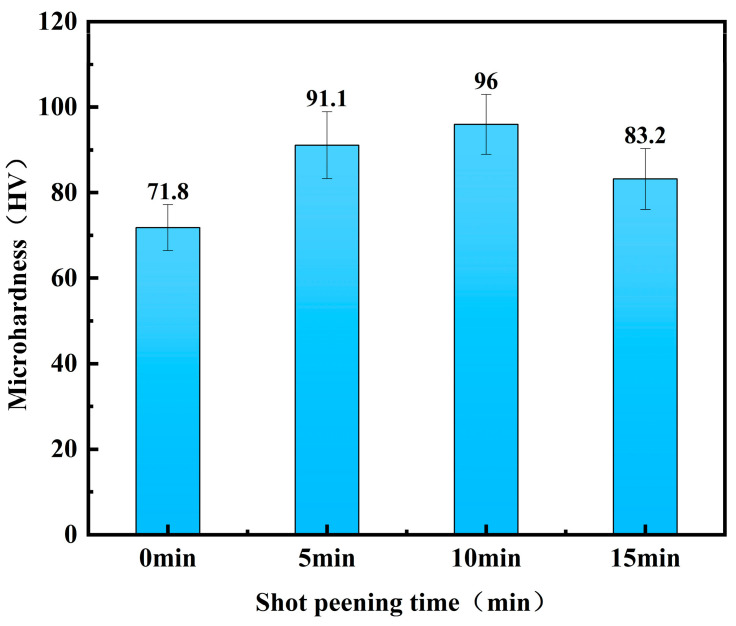
Variation in microhardness of the 7075 aluminium alloy under different shot peening times.

Hardness is a key indicator for evaluating the effectiveness of shot peening [29], and significant variations were observed in this study. Figure 2 shows the variation in the microhardness of the 7075 aluminium alloy at different shot peening times. The experimental results showed that shot peening is effective in increasing the hardness within a certain time range compared with gunshot-peened 7075 aluminium alloy. The hardness value of the gunshot-peened specimen was 71.8 HV; when the shot peening time was 5 min, the hardness increased to 91.1 HV; when the shot peening time was extended to 10 min, the hardness value reached a maximum of 96.0 HV; however, when the shot peening time was further increased to 15 min, the hardness decreased back to 82.2 HV. Compared with the original specimen, the hardness value was significantly improved. With the increase in shot peening time, the surface hardness of the 7075 aluminium alloy showed a trend of increasing and then decreasing, but the hardness did not increase continuously with the increase in shot peening time [32], indicating that the shot peening time has a limited effect on the thickness of the hardened layer. When the intensity of shot peening is certain, the degree of hardening reaches its peak, and extension of the shot peening time will heat up the surface of the specimen and release the internal stresses. Ultimately, the strain layer will reach a dynamic equilibrium, and the thickness of the hardened layer will no longer increase [33,34,35].

**Figure 3:**
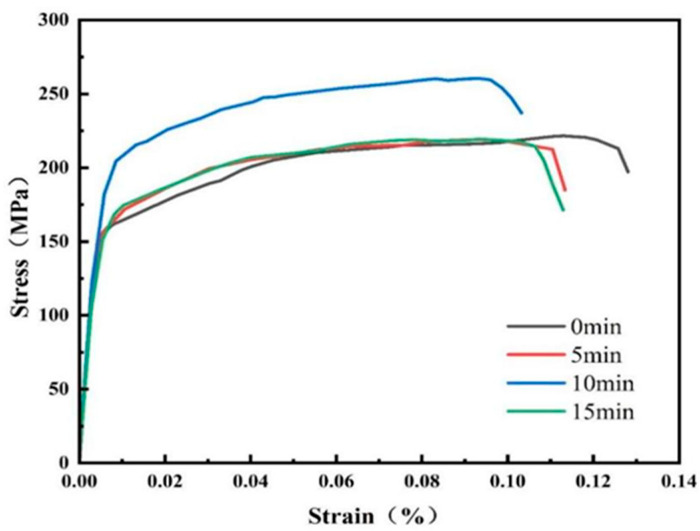
Stress–strain curve of the 7075 aluminium alloy.

As shown in Figure 3, with an increase in shot peening time, the tensile strength increased and then decreased, and the elongation decreased and then increased. In the elastic region, the 95% confidence interval of stress was within ±5 MPa. As the strain increased into plastic deformation, the intervals widened to ±10 MPa. This reflected microstructural variability during strain hardening, but the intervals remained within 6% of the mean, confirming acceptable data consistency. As presented in Table 3, at a shot peening time of 5 min, the elongation of the specimen decreased to 11.3%, and the change in strength was not significant. When the shot peening time was further extended to 10 min, the strength of the material significantly increased, whereas the elongation significantly decreased. When the shot peening time was extended to 15 min, the strength of the material significantly decreased. A comparison of the results at different shot peening times showed that the strength of the 7075 aluminium alloy increased and then decreased with extension of the shot peening time, whereas the elongation decreased and then increased. Further analysis showed that shot peening improved the surface strength of the material by introducing residual compressive stresses and refining the grains; however, it also increased the brittleness of the material.

Ten min was the optimal shot peening parameter, balancing strength improvement and hardness enhancement. Shot peening changed the mechanical properties in accordance with the literature [36].

### 3.2. Tensile Fracture Morphology Analysis

**Figure 4:**
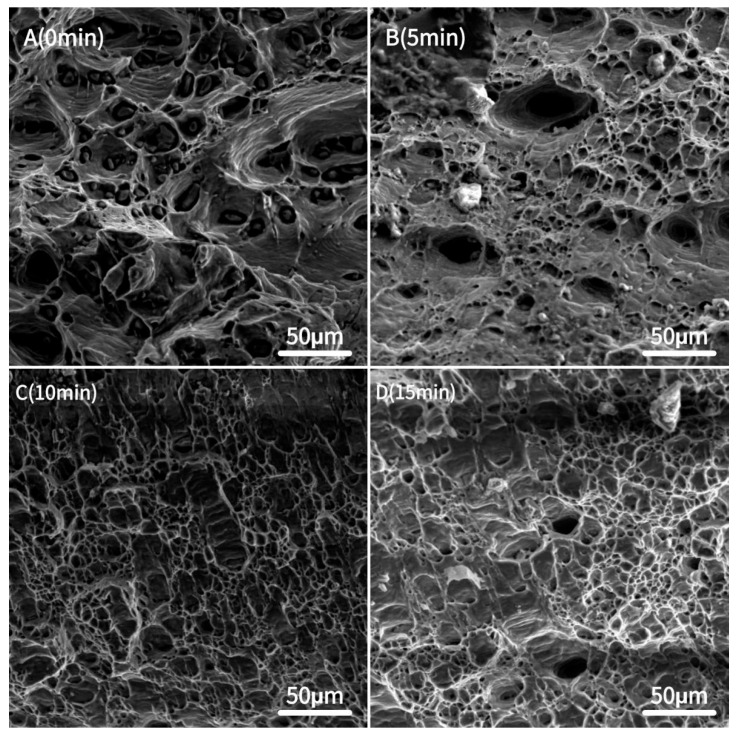
Tensile fracture morphology of the 7075 aluminium alloy.

Figure 4 shows the change in fracture morphology of the 7075 aluminium alloy under different shot peening times. Without shot peening, the fracture surface of the 7075 aluminium alloy showed a typical tough nest structure, indicating that the fracture mode of the material was mainly a toughness fracture. The size and distribution of the tough nests were relatively uniform, and the material experienced a large plastic deformation before fracture. After shot peening for 5 min, the ductile nests on the fracture surface became shallower and less numerous, and some small cracks and holes began to appear, indicating that the shot peening process had caused some hardening and stress concentration on the material surface. After 10 min of shot peening, the tough nests on the fracture surface were further reduced and more small cracks and holes appeared. The appearance of these cracks and holes indicated that the hardening of the material surface increased further and the toughness of the material started to decrease. After 15 min of shot peening, the number of tough nests on the fracture surface increased and their depth and distribution became more uniform, indicating that the toughness of the material had recovered.

As the shot peening time increased, the fracture microstructure of the 7075 aluminium alloy transitioned from ductile to brittle fracture. Notably, after 15 min of shot peening, the material exhibited improved toughness and increased elongation. This indicated that while shot peening enhanced the surface hardness of the material, a prolonged shot peening duration may have induced structural changes within the material, ultimately affecting its fracture behaviour [34].

### 3.3. Three-Dimensional Morphology Analysis

**Figure 5:**
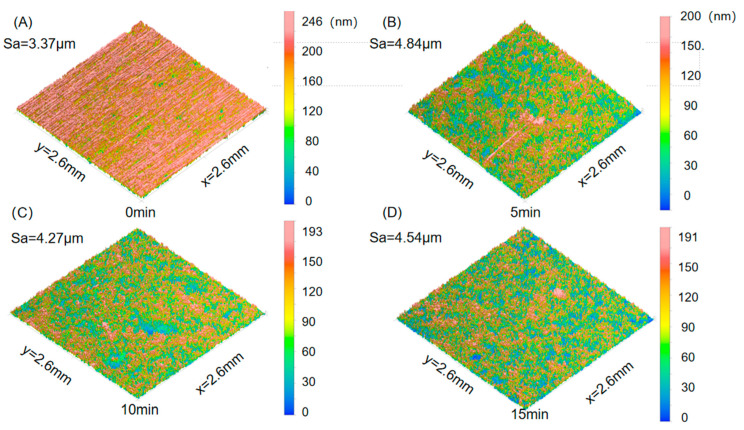
Three-dimensional surface morphology of the 7075 aluminium alloy surface: (**A**) 0 min, (**B**) 5 min, (**C**) 10 min, (**D**) 15 min.

**Figure 6:**
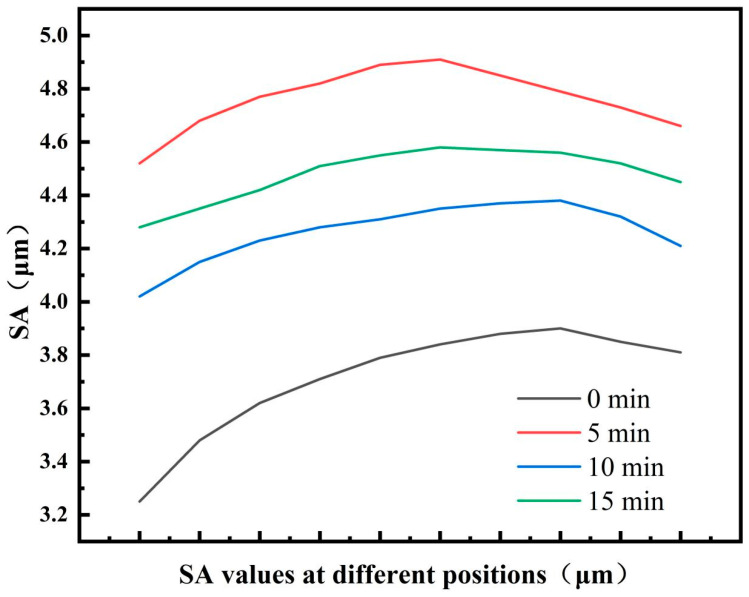
Surface roughness (Sa) parameters of the 7075 aluminium alloy surface.

Figure 5 shows the surface roughness morphology of the 7075-O aluminium alloy specimens after different shot peening times. During shot peening, the high-speed impact of the projectile on the surface of the material causes the formation of rough peaks and valleys on the contact surface, which in turn changes the surface morphology of the material. Figure 6 shows the variation in the surface roughness parameters of the material at different shot peening times, where Sa is the roughness of the shot-peened surface. The Sa value of the specimen without shot peening was 3.37 μm, and when the shot peening time reached 5 min, the Sa value reached 4.84 μm and the surface roughness of the specimen reached the highest value. When the shot peening time increased to 10 min, Sa decreased to 4.27 μm, indicating that the surface roughness of the specimen decreased when the shot peening time was excessively long. When the shot peening time reached 15 min, the Sa value was 4.54 μm. With the increase in shot peening time, the surface roughness showed a trend of first increasing, then decreasing, and then increasing again. Therefore, by reasonably controlling the shot peening time, the ideal surface roughness can be obtained, thus providing a material with good comprehensive mechanical properties. Shot peening introduces a gradient residual compressive stress layer on the material surface through high-speed shot impact, which is the core mechanism for enhancing material hardness and tensile strength [29]. As shown in Figure 6, when the shot peening time was 5 min, the surface roughness (Sa = 4.84 μm) significantly increased, indicating that plastic deformation exacerbated surface irregularities, and at this point, the residual compressive stress layer began to form but was unevenly distributed. With the shot peening time extended to 10 min, the surface roughness decreased to 4.27 μm, and the microstructure became more uniform, with optimised residual stress distribution, which inhibited crack initiation and slowed down crack propagation, thus significantly improving hardness and tensile strength. However, when the shot peening time was too long, excessive plastic deformation led to thermal effect accumulation, causing relaxation of residual stress, and the roughness recovered to 4.54 μm, leading to a decrease in hardness and strength [30]. The shot peening time should be controlled at the critical point between residual stress strengthening and thermal softening effects to achieve optimal performance. Additionally, the increase in surface roughness during the initial stage of shot peening (5 min) introduced micro-defects and reduced toughness, while a moderate shot peening time (10 min) partially restored material toughness through grain refinement and micro-defect compaction [36,37].

### 3.4. DIC-Based Damage Evolution Analysis

The experimental equipment was manufactured by Correlated Solutions, model number VIC-3D. The equipment supports 3D full-field strain measurement.

**Figure 7:**
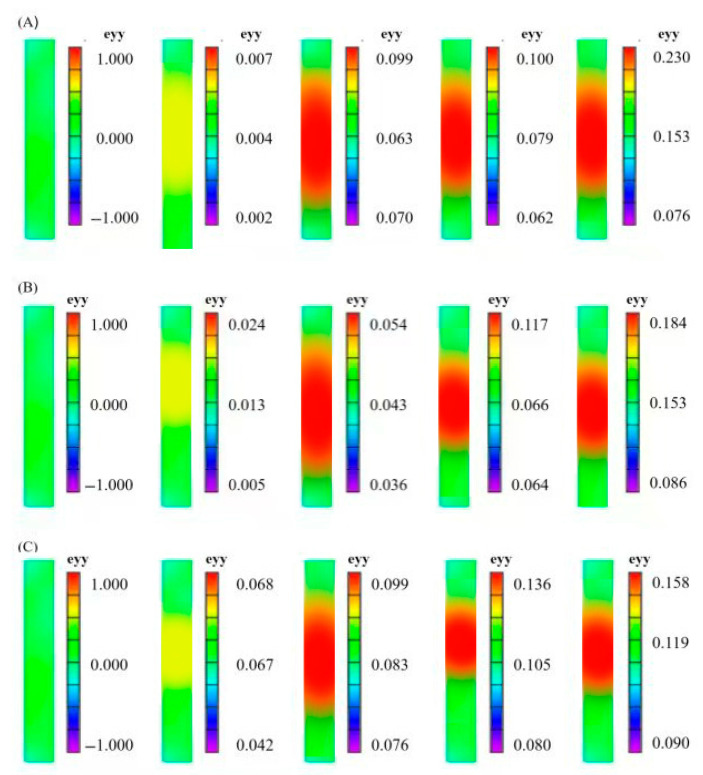

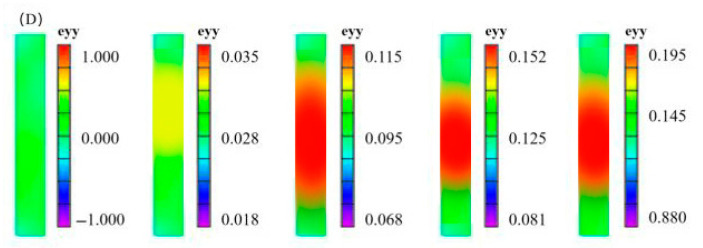
The strain in the Y-direction: (**A**) 0 min, (**B**) 5 min, (**C**) 10 min, (**D**) 15 min.

Figure 7 shows the strain cloud diagram of the specimens under different shot peening times. Unagreed Sample (A): The strain distribution was relatively uniform, with no significant concentration areas in the early plastic stage. The maximum strain before fracture was concentrated in the middle of the gauge length. 5-min Shot Peening (B): A residual compressive stress layer formed on the surface, leading to a more dispersed early strain distribution. However, multiple local strain concentration points appeared during the plastic stage, indicating that micro-defects introduced by shot peening exacerbated local deformation [34]. 10-min Shot Peening (C): Under optimal shot peening time, the strain concentration area significantly decreased, and high-strain zones concentrated in a single narrow band, corresponding to the highest tensile strength (260 MPa) and lowest elongation (10.3%) in Table 3, confirming the positive role of residual stress homogenisation in controlling deformation [29]. 15-min Shot Peening (D): Excessive shot peening led to surface stress relaxation, restoring a more dispersed strain distribution (similar to the unagreed sample), but the number of local strain concentration points increased, consistent with the decrease in hardness and the increase in elongation (11.2%) [30]. As can be observed, with the increase in deformation, the 7075 aluminium alloy was in an obvious plastic stage, and the specimen began to exhibit a strain concentration area and local deformation. In the early stages of specimen stretching, the material was in the elastic stage, the specimen was mainly deformed uniformly, and the deformation was small. As the load increased, the specimen entered the plastic deformation stage and a strain concentration phenomenon occurred. As the tensile load continued to increase, the specimen entered the local deformation stage. The shot-peened specimens exhibited a more uniform strain distribution during the tensile process and a more complex strain concentration phenomenon. With an increase in shot peening time, the strain concentration area on the specimen surface became more significant, and the strain value decreased.

The rapid damage evolution of the 10 min shot peening sample was closely related to the residual stress gradient distribution and micro-defect evolution. The residual compressive stress layer introduced by shot peening gradually released under tensile loading, forming a dislocation slip concentration driven by a stress gradient. When the shot peening time was 10 min, the surface grains refined to the nanoscale, and dislocation movement was hindered by grain boundaries, leading to rapid accumulation of local strain energy. The DIC strain cloud map shows a narrow high-strain zone reflecting the nucleation of micro-cracks caused by dislocation pile-up, which can be explained by the following mechanisms: 1. Residual stress release and dislocation proliferation: The compressive stress introduced by shot peening is partially released during the initial tensile stage, promoting dislocation source activation. In the 10 min shot peening sample, the shortened dislocation slip distance due to grain refinement led to a rapid increase in dislocation density in local areas, forming strain concentration. 2. Dynamic equilibrium of micro-defects: The rough surface (Sa = 4.84 μm) introduced during the early shot peening phase (5 min) introduces micro-cracks, but at 10 min, grain refinement and residual stress homogenisation partially close surface defects (Sa decreases to 4.27 μm), instead exacerbating internal dislocation entanglement and accelerating damage accumulation.

An average strain factor was employed to quantitatively investigate the apparent damage evolution behaviour of the 7075 aluminium alloy under varying shot peening times. On the surface strain cloud map of the 7075 aluminium alloy during the tensile process, 1000 data points were randomly selected from the entire deformation region of the specimen, and it was ensured that these data points completely covered the entire region of the specimen. Simultaneously, 300 data points were randomly selected in the deformation concentration area to ensure that these data points completely covered the deformation concentration area. The difference between the strain values of 300 scattered spots in different regions and the average strain value of the 1000 scattered spots in the whole region was used to express the average strain factor $\bar{\varepsilon }$ .

(1) $$\bar{\varepsilon }=|\frac{1}{300}\sum_{i=1}^{300}\;(\varepsilon_{yy}{)}_{i}-\frac{1}{1000}\sum_{i=1}^{1000}\;\varepsilon_{yy}{)}_{i}|\;$$

where $\frac{1}{300}\sum_{i=1}^{300}\;(\varepsilon_{yy}{)}_{i}$ represents the average strain value of 300 data points in the area of deformation concentration, and $\frac{1}{1000}\sum_{i=1}^{1000}\;{\left(\varepsilon_{yy}\right)}_{i}$ represents the average strain value of 1000 data points over the entire deformation region of the specimen.

**Figure 8:**
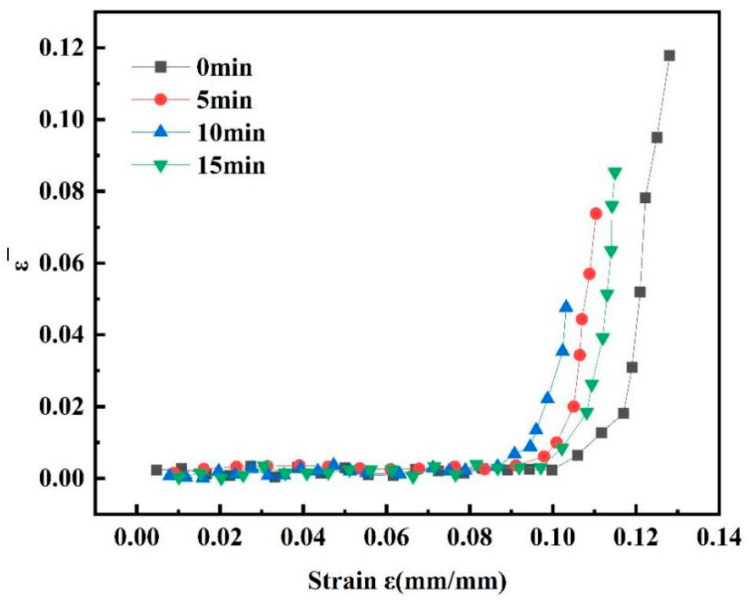
Variation curve of the average strain factor of the 7075 aluminium alloy.

To determine the effects of various shot peening durations on the tensile damage progression of the 7075 aluminium alloy, graphs were plotted depicting the average strain factor against macroscopic strain for specimens subjected to different shot peening times. As is evident from Figure 8, during the initial loading phase, the average strain factor exhibited a gradual rise with increasing macro-strain. Upon reaching the local deformation stage, however, the average strain factor surged, marked by a pronounced inflection point in the curve.

The more significant the local deformation, the higher is the average strain factor and, accordingly, the more serious is the damage to the alloy. Therefore, the average strain factor can serve as a crucial indicator for characterising the extent of damage in alloys. According to the Lemaitre damage theory, internal damage accumulates within a material when it is subjected to an external load. Damage can be quantified using specific variables. Consequently, to characterise the damage degree of the alloy, it is essential to consider these factors. The damage factor $D_{\varepsilon }$ is defined as follows:

(2) $$D_{\varepsilon }=\bar{\varepsilon }/{\bar{\varepsilon }}_{max}$$

In the above equation, ${\bar{\varepsilon }}_{max\;}$ represents the average strain factor at fracture of the alloy,  ε is the maximum value that can be achieved, and $D_{\varepsilon }$ denotes the damage factor.

**Figure 9:**
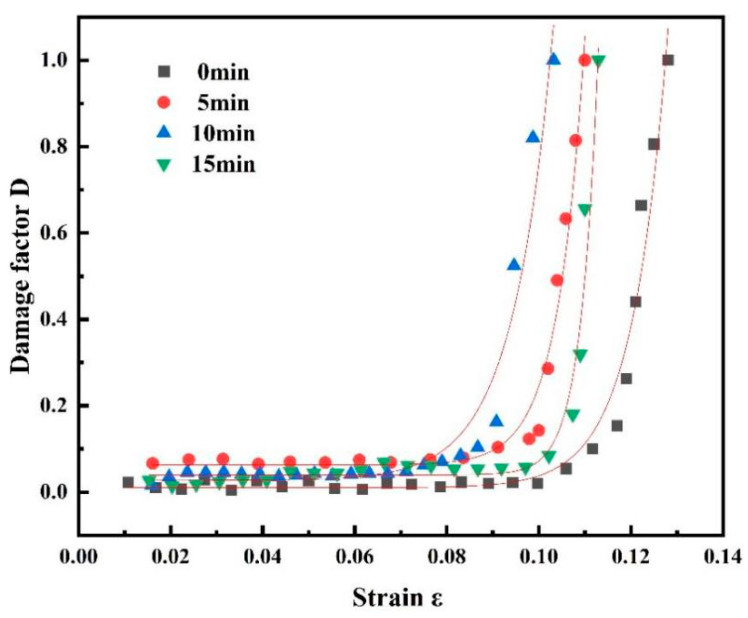
Evolution curve of damage factor for the 7075 aluminium alloy.

Figure 9 shows the evolution curve of the damage factor, which shows that the rate of damage accumulation with an increase in shot peening time first increased and then decreased, indicating that the shot peening treatment simultaneously enhanced the surface strength of the material and exacerbated the early emergence of internal damage. The value of the damage variable D ranged from 0 to 1. When D = 0, there was no damage to the material, and the 7075 aluminium alloy was in the initial state of integrity; when D was between 0 and 1, the 7075 aluminium alloy was in the process of tensile stretching, and at this time, the damage of the 7075 aluminium alloy increased with an increase in D. When D = 1, the 7075 aluminium alloy was completely damaged and had lost its load-bearing capacity. With an increase in shot peening time, the rapid damage stage of the material appeared earlier. When the shot peening time was 15 min, rapid damage began to occur with a lag.

The 7075 aluminium alloy damage factor was fitted using the function to obtain the 7075 aluminium alloy damage evolution equation:

(3) $$0\;\min:\;D=0.00996+\left(6.69\times 10^{-8}\right)e^{\frac{\varepsilon }{0.00772}}$$

(4) $$5\;\min:\;D=0.0626+\left(8.89\times 10^{-6}\right)e^{\frac{\varepsilon }{0.00592}}$$

(5) $$10\;\min:\;D=0.0270+\left(8.89\times 10^{-6}\right)e^{\frac{\varepsilon }{0.00883}}$$

(6) $$15\;\min:\;D=0.0398+\left(1.11\times 10^{-13}\right)e^{\frac{\varepsilon }{0.00379}}$$

Uniform substitution of the fitted equations yielded the general equation: $D=y_{0}+A_{1}e^{\frac{-\varepsilon }{t_{1}}}$ .

Here, $y_{0}$ , *A*_1_, *t*_1_: model parameters; ε : material strain; and *D*: damage factor.

Table 4 shows the fitting parameters for each equation.

### 3.5. Damage Evolution Analysis Based on Infrared Thermal Imaging

To quantitatively investigate the damage evolution behaviour of the 7075 aluminium alloy under various shot peening times, an average temperature factor was introduced. On the infrared thermal imaging temperature distribution map of the 7075 aluminium alloy during the tensile process, 100 data points are randomly selected in the entire deformation region of the specimen, ensuring that these data points completely covered this region. Simultaneously, 20 data points were randomly selected from the deformation concentration area to ensure that they completely covered the deformation concentration area. The difference between the temperature values of the 20 data points in different regions and the average temperature of the 100 data points in the entire region was used to express the average temperature factor $\bar{T}$ .

To quantitatively characterise the apparent damage evolution behaviour of the 7075 aluminium alloy at different shot peening times, an average temperature factor, $\bar{T}$ , was introduced. The formula is expressed as follows:

(7) $$\bar{T}=\left|\frac{1}{20}\sum_{i=1}^{300}\left(T_{i}\right)-\frac{1}{100}\sum_{i=1}^{1000}\left(T_{i}\right)\right|\;$$

Here, $T_{i}$ is the temperature of the i-th data point of the specimen in the infrared thermal imaging experiment. Twenty data points were randomly selected from areas of concentrated deformation to ensure coverage of the area, and 100 data points were randomly selected from the overall deformation area of the specimen to ensure the coverage of the entire area.

On this basis, to characterise the degree of damage to the 7075 aluminium alloy, the damage factor $D_{T}$ was defined as follows:

(8) $$D_{T}=\bar{T}/T_{max}$$

In the above equation, ${\bar{T}}_{max}$ represents the maximum value that can be reached by the average temperature factor at alloy fracture, whereas $D_{T}$ denotes the damage factor.

The above image was captured by an infrared thermal imaging camera, model FLIR T420, with a frame rate of 60 Hz.

**Figure 10:**
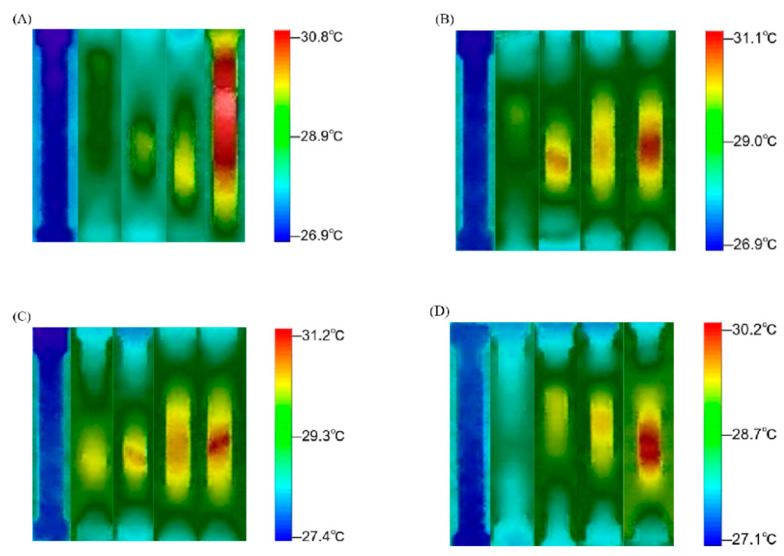
Evolution of temperature field during stretching of the 7075 aluminium alloy: (**A**) 0 min, (**B**) 5 min, (**C**) 10 min, (**D**) 15 min.

To study the damage evolution law during material deformation, the tensile test process was monitored using an infrared thermal camera. Thermal distribution profiles across specimen surfaces under varying shot peening durations are delineated in Figure 10. As can be observed in the figure, when the specimen was subjected to a tensile load, the temperature change on the surface of the alloy during the fracture process underwent a gradual warming process. A more intense deformation region corresponded to a higher temperature value, and there was a certain correlation between the temperature and strain distributions. In the elastic deformation stage, the temperature did not change much and fluctuated between 26.9 °C and 27.4 °C. The damage was still in the embryonic stage, the stress distribution was uniform, and it proceeded slowly. When the specimen entered the plastic stage, the temperature showed an obvious rising trend, and the regional temperature reached 30.2–31.2 °C. According to the theory of heat conduction, we can analyse the relationship between heat aggregation and damage expansion to locate the damage accumulation area, which is closely related to the plastic power dissipation and microstructural changes of the material. Infrared thermography showed that the 10 min shot-peened sample exhibited localised high-temperature zones during the plastic stage, with their thermal distribution highly coinciding with the DIC strain concentration areas. This thermal–hydraulic coupling effect can be explained through the following processes: 1. Plastic work dissipation: During dislocation slip and twinning, approximately 90% of mechanical energy is converted into heat [28]. Due to the high dislocation density in the 10 min shot-peened sample, the rate of plastic work dissipation accelerated, leading to significant local temperature increases. 2. Residual stress thermal relaxation: The lattice distortion energy caused by shot peening is released through thermal activation during tensile testing, further exacerbating the temperature rise. The high-temperature zone in Figure 10C was located in the centre of the sample, consistent with the region where residual compressive stress relaxation led to dislocation recombination.

**Figure 11:**
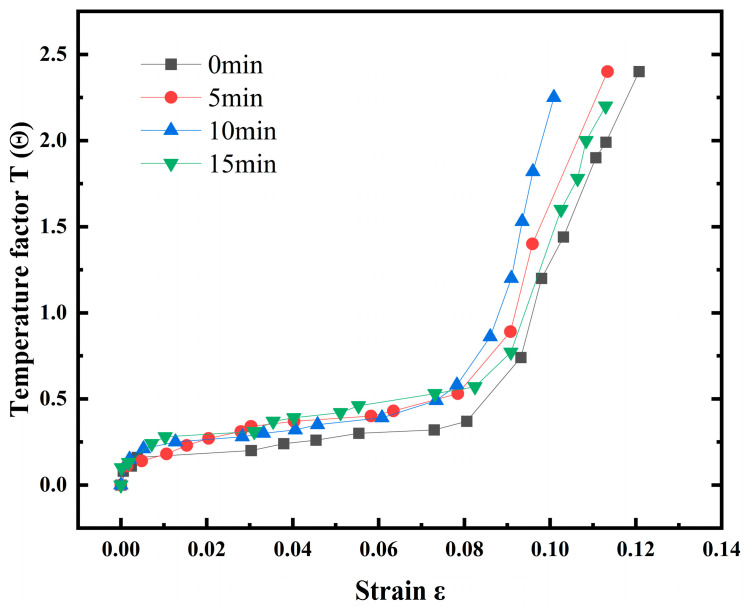
Evolution curve of average temperature factor for the 7075 aluminium alloy.

Figure 11 shows the evolution curves of the temperature factor of the 7075 aluminium alloy at different shot peening times. With an increase in plastic deformation, the temperature inside the material rose faster, especially after 10 min of shot peening, when a rapid temperature rise occurred. The longer the shot peening time, the earlier the rapid temperature rise phase of the material occurred, and the rapid temperature rise phase started to occur with a lag when the shot peening time was 15 min.

**Figure 12:**
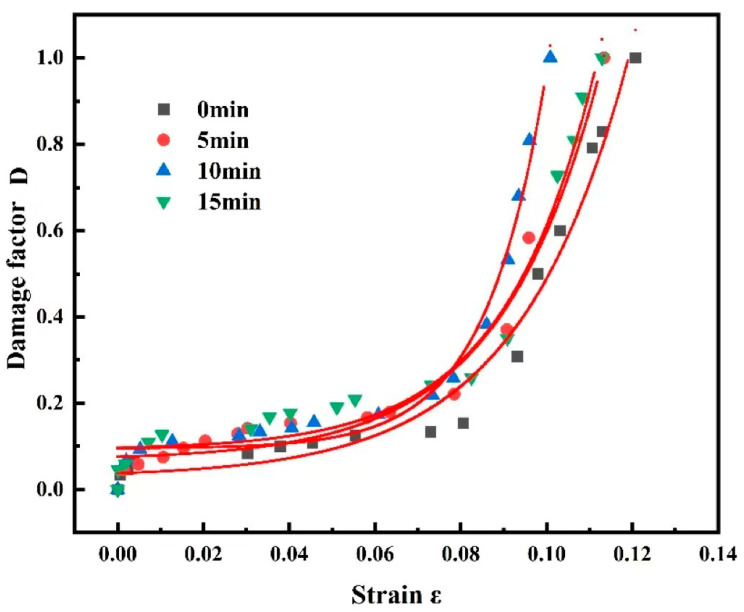
Evolution curve of the temperature damage factor for the 7075 aluminium alloy.

Figure 12 shows the evolution curve of the temperature damage factor, which indicates that the damage to the alloy still exhibited two stages: smooth development and rapid damage. With an increase in plastic deformation, the damage reached a critical value, and then rapid damage started. The longer the shot peening time, the earlier appeared the rapid damage. When the shot peening time was increased to 15 min, the rapid damage appeared to lag. This indicated that shot peening exacerbated the early sprouting of internal damage while enhancing the surface strength of the material. This phenomenon may be attributed to the combination of internal stress release at the surface of the material owing to the shot peening effect and reorganisation of the dislocation structure, which reduced the brittleness of the material and increased its ductility, leading to a lag in the rapid damage stage [37,38,39].

The 7075 aluminium alloy damage factor was fitted using the function to obtain the 7075 aluminium alloy damage evolution equation:

(9) $$0\;\min:\;y=0.0285+0.00923e^{\frac{\varepsilon }{0.02558}}$$

(10) $$5\;\min:\;y=0.0685+0.00737e^{\frac{\varepsilon }{0.00123}}$$

(11) $$10\;\min:\;y=0.0950+7.33\times 10^{-4}e^{\frac{\varepsilon }{0.01411}}$$

(12) $$15\;\min:\;y=0.0919+0.00498e^{\frac{\varepsilon }{0.02151}}$$

Uniform substitution of the fitted equations yielded the general equation: $D=y_{0}+A_{1}e^{\frac{-\varepsilon }{t_{1}}}$ .

Here, $y_{0}$ , ${\;A}_{1}$ , $\;t_{1}$ : model parameters; ε : material strain; and *D*: damage factor.

Table 5 shows the fitting parameters for each equation.

### 3.6. Comparative Analysis of the Two Damage Factors

**Figure 13:**
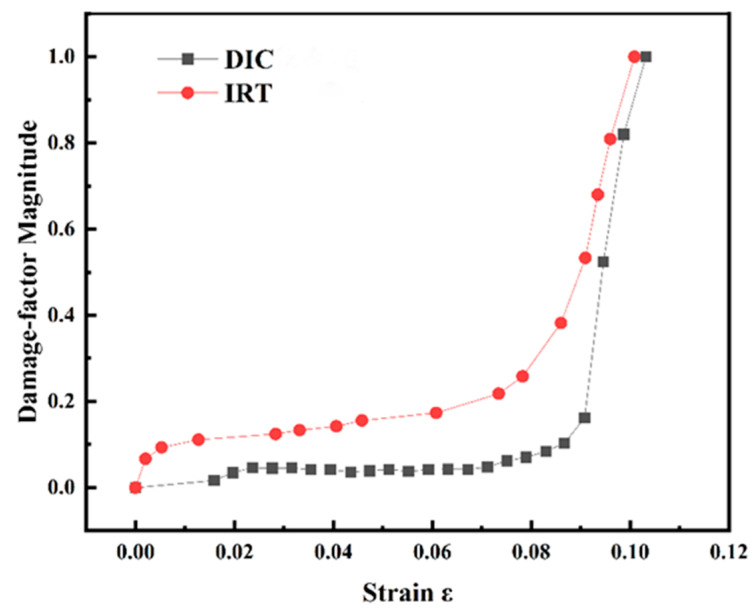
Comparison curve of the evolution of the two damage factors of the 7075 aluminium alloy after 10 min of shot peening.

Figure 13 shows the comparison curves of the evolution of the two damage factors of the 7075 aluminium alloy after 10 min of shot peening. In the early stages of tensile stress, the damage factors of the DIC and IRT methods showed a gently increasing trend, indicating that the material was in the stage of smooth damage accumulation. After the damage surpassed a critical value, the damage factors of both methods exhibited exponential growth, corresponding to a rapid damage stage. Under the same shot peening time, the damage evolution patterns characterised by the two methods were similar to the increase in plastic deformation, showing good consistency. The critical damage values in the two damage evolution curves were different, and the IRT critical damage value was larger than the DIC critical damage value. This was primarily because the DIC technique captured the strain distribution on the surface of the material, whereas the IRT technique reflected the energy dissipation process inside the material through the temperature change. During the stretching process, the temperature of the surface strain concentration area increased faster, whereas the energy dissipation process of the internal damage lagged behind, resulting in a larger critical damage value as measured by the IRT technique [28].

## 4. Conclusions

To elucidate the influence of shot peening duration on the critical characteristics of 7075-T6 aluminium alloy, this research subjected specimens to mechanical shot blasting under varying exposure times. Comprehensive examinations were conducted regarding microhardness evolution, topographical profiles, tensile response behaviours, damage progression mechanisms, and fractographic features, yielding the following principal findings:

Prolonged shot peening duration induced a non-monotonic response in the 7075-T6 alloy’s mechanical properties: both surface hardness and ultimate tensile strength demonstrated initial enhancement followed by reduction, while elongation conversely exhibited an initial decline prior to recovery. Optimal strength characteristics were attained at 10 min shot peening exposure, where peak microhardness and tensile resistance coincided with moderate ductility reduction.

After shot peening, the surface roughness of the material increased significantly. With the increase in shot peening time, the surface roughness first increased and then decreased. Reasonable control of shot peening time can effectively optimise the surface roughness of the material and improve its comprehensive mechanical properties. The shot peening time can be controlled to balance the roughness and internal stress, thereby enhancing the comprehensive mechanical properties by inhibiting crack propagation.

Analysis based on temperature evolution and DIC showed that shot peening improved the tensile strength of the material, shortened the critical strain for rapid damage occurrence, and accelerated the damage accumulation rate. With a shot peening time of 10 min, a rapid damage stage first appeared compared with the other shot peening time specimens. The trends in the parameters of the damage evolution equations of DIC and IRT were consistent, and the IRT critical damage value was larger than the DIC critical damage value. The shot peening time influenced the damage evolution rate by regulating the competition between residual stress release and dislocation movement. The 10 min shot peening treatment formed a nanocrystalline layer and high residual compressive stress on the surface, both of which limited dislocation slip and localised strain energy, thus accelerating damage accumulation. However, an excessively long shot peening time (15 min) led to thermal effects that caused stress relaxation and grain boundary weakening, partially restoring dislocation mobility, which slowed down the damage evolution (the $D_{\varepsilon }$ curves in Figure 10 showed a lag).

This study successfully constructed a real-time health monitoring system for materials using multi-physics field coupling analysis technology, providing an innovative solution for predicting material failure in extreme environments.

## Figures and Tables

**Table 1:** Chemical composition of alloy 7075 (mass fraction, %).

Si	Fe	Cu	Mg	Zn	Ti	Mn	Cr	Al
0.02	0.163	1.662	2.605	5.829	0.022	0.02	0.192	Bal

**Table 2:** Shot peening process.

Lab Number	Shot Peening Time/(min)	Specimen Thickness/(mm)
1	0	1.52
2	5	1.51
3	10	1.51
4	15	1.50

**Table 3:** Mechanical properties of the 7075 aluminium alloy.

Lab Number	Tensile Strength (MPa)	Elongation Rate (%)	Hardness (HV)
0 min	221	12.8	71.8
5 min	219	11.3	91.1
10 min	260	10.3	96.0
15 min	218	11.2	83.2

**Table 4:** Parameter list of damage factor fitting equations for different shot peening times.

RATIO	0 min	5 min	10 min	15 min
$y_{0}$	0.00996	0.0626	0.0270	0.0398
$A_{1}$	6.69 × 10^−8^	8.89 × 10^−6^	8.89 × 10^−6^	1.11 × 10^−13^
$t_{1}$	−0.00772	−0.00592	−0.00883	−0.00379

**Table 5:** Parameter list of damage factor fitting equations for different shot peening times.

RATIO	0 min	5 min	10 min	15 min
$y_{0}$	0.0285	0.0685	0.0950	0.0919
$A_{1}$	0.00923	0.00737	7.33 × 10^−4^	0.00498
$t_{1}$	−0.02558	−0.0234	−0.01411	−0.02151

## Data Availability

The original contributions presented in this study are included in the article. Further inquiries can be directed to the corresponding author.
